# Effect of Premedication with Indomethacin and Ibuprofen on Postoperative Endodontic Pain: A Clinical Trial

**DOI:** 10.7508/iej.2016.01.011

**Published:** 2015-12-24

**Authors:** Fatemeh Mokhtari, Kamal Yazdi, Amir Mohammad Mahabadi, Seyed Jalil Modaresi, Zeinab Hamzeheil

**Affiliations:** a *Department of Endodontics, Shahid Sadoughi University of Medical Sciences, Yazd, Iran****; ***; b * General Dentist, Tehran, Iran*

**Keywords:** Ibuprofen, Indomethacin, Irreversible Pulpitis, Non-Steroidal Anti-Inflammatory Drugs, Post-Endodontic Pain

## Abstract

**Introduction:**

Post-endodontic pain is one of the main problems for both patients and dentists. The purpose of this study was to compare the effectiveness of premedication with indomethacin and ibuprofen for management of postoperative endodontic pain.

**Methods and Materials:**

In this clinical trial, mandibular molars with irreversible pulpitis were endodontically treated in 66 patients. The medicines were prepared similarly in the form of capsules containing 400 mg ibuprofen (group A), 25 mg indomethacin (group B) and placebo (group C). The patients were given one capsule 1 h before the start of treatment. Patients recorded their pain measured by a visual analogue scale (VAS) at medication time, during treatment and 8, 12 and 24 h after treatment. The data were analyzed using the chi-square, repeated measures ANOVA, paired t-test, Tamhane and Pearson correlation coefficient.

**Results:**

Ibuprofen and indomethacin significantly reduced the postoperative pain in comparison with placebo during treatment and 8 h after treatment; however, there were no significant differences between them 12 and 24 h after treatment.

**Conclusion:**

Premedication with ibuprofen and indomethacin can effectively control short term post-operative pain; the lower incidence of side effects and greater analgesic power of ibuprofen make it a superior choice.

## Introduction

Appropriate anesthesia and successful control of postoperative endodontic pain is one of the major challenges in endodontic treatment specially in teeth with irreversible pulpitis [1]. Despite the advances in root canal treatment and increase in the awareness about periapical inflammation and pulpitis, postoperative endodontic pain can be a major problem for both patient and dentist. More than 40% of endodontic patients reported various degrees of pain after endodontic treatment [2, 3]. Generally postoperative endodontic pain is contributed to the inflammatory mediators that activate sensitive nociceptors and lead to central and peripheral hyperalgesia mechanisms [4]. Among inflammatory mediators, prostaglandins have crucial function in pathogenesis of periradicular and pulpal diseases [5].

Non-steroidal anti-inflammatory drugs (NSAIDs) such as ibuprofen and indomethacin are effective in reducing the pain in endodontic treatments and are commonly prescribed for this reason [6]. Studies show that analgesic and anti-inflammatory effect of NSAIDs has been caused by inhibiting cyclooxygenase pathway and subsequently reduce the pain inducing role of arachidonic acid metabolites such as prostaglandin and thromboxane [7]. 

Nevertheless recent investigations propose that this class of drugs has other effects embracing inhibition of cytokine synthesis or major cellular signaling pathways of inflammatory responses [7-9]. Rudimentary investigations prove that premedication with NSAIDs such as flurbiprofen, ibuprofen, tenoxicam, or rofecoxib can decrease the amount of post-operative pain more efficiently than placebo [10-12]. 

Premedication with NSAIDs will diminish the inflammatory process before its onset in endodontic treatment [13]. Gopikrishna *et al. *[11] showed that single dose prophylactic prescription of rofecoxib (50 mg) or ibuprofen tablet (600 mg) significantly relieves the postoperative endodontic pain. In another study by Arslan *et al*. [12] prophylactic prescription of ibuprofen (200 mg) and tenoxicam (20 mg) significantly reduced the postoperative endodontic pain. However, Attar *et al*. [14] has shown that there was no significant difference in postoperative endodontic pain between patients who took ibuprofen tablet, ibuprofen liquigel (400 mg) and placebo, preoperatively.

Indomethacin is not routinely prescribed for endodontic treatment but because of its extensive anti-inflammatory effect, it is commonly used to relieve muscle and joint pain [15]. Likewise Parirokh *et al*. [16] demonstrated the effectiveness of indomethacin and ibuprofen on increasing the depth of inferior alveolar nerve block. However, many studies have evaluated the effectiveness of prophylactic premedication with ibuprofen on postoperative endodontic treatment [11, 12, 14]. 

No study has compared the effectiveness of prophylactic premedication with ibuprofen and indomethacin on pain relieve after endodontic procedures. Therefore the aim of this clinical trial was to compare the effects of premedication with indomethacin and ibuprofen on postoperative endodontic pain.

## Materials and Methods

A total of 66 patients aging 19 to 30 years were chosen based on the results of the pilot study to achieve 95% confidence interval (two-side) and 90% power (*n*=22). Samples were selected randomly by table of random numbers in each three groups for this double-blind, placebo-controlled, parallel-group, single-dose randomized controlled clinical trial. Each patient consented to a protocol reviewed and approved by the Medical Ethics Committee of Shahid Sadoughi University of Medical Sciences, Yazd, Iran (Grant No.: 46210). 

At first, patients completed a quantitative questionnaire and marked the severity of their pretreatment pain on a 100-mm visual analogous scale (VAS).

Inclusion and exclusion criteria were considered for this study. Patients who had first or second mandibular molars with irreversible pulpitis and reported spontaneous pain at least 30 mm on VAS, with normal radiographic view, without any lesion or sinus tract (acute periapical abscess) and exhibit a long response to electric pulp testing (Analytic Technology, Redmond, WA, USA) and cold testing with Endo-Ice frozen gas (Coltene/Whaledent Inc., Mahwah, NJ, USA), were included. Patients had to complete and sign an informed consent.

Volunteers were excluded if they were younger than 17 years of age and older than 50, if they took any analgesic during the last 12 h or used to consume medicines interfering with NSAIDs, if they were allergic to NSAIDs or lidocaine or had systemic disease, if were in pregnancy or nursing phase, if they had teeth with periapical lesion or acute periapical abscess, aggressive periodontal disease, or non-restorable/previously treated teeth, or had pain in more than one tooth, and if they failed to comprehend the protocol of the study or sign the informed consent.

Determination of pulp status was done according to dental history, clinical findings and radiographic signs. Patients were randomly assigned to 3 equal groups (*n*=22). With a double blind design, medicines were administered in all groups 1 h before endodontic procedure; group A [400 mg ibuprofen tablet (Hakim Pharmacy Co, Tehran, Iran)], group B [25 mg indomethacin (Hakim Pharmacy Co, Tehran, Iran) and group C (placebo, starch powder) which were all prepared in similar capsules. The dentist and patients were not aware of the capsule contents (double-blind design).

Inferior alveolar nerve block injection was done using 1.8 mL of 2% lidocaine with 1:80000 epinephrine (Darupakhsh, Tehran, Iran). Ten min later, preoperative pain was recorded on VAS. The teeth were isolated using rubber dam and access cavities were prepared. Working length was determined with Root ZX apex locator (J. Morita USA, Inc., Irvine, CA, USA) and confirmed with periapical radiographs. Cleaning and shaping was done using the passive step-back technique.

Normal saline and 2% sodium hypochlorite were used as irrigants. Then canals were dried with paper points and obturation of the canals was accomplished by gutta-percha (Gapadent Co Ltd, Tianjin, China) and AH-26 sealer (Dentsply, Tulsa Dental, Tulsa, OK, USA) using the lateral condensation technique within 0.5-0.1 mm of radiographic apex. 

**Figure 1:**
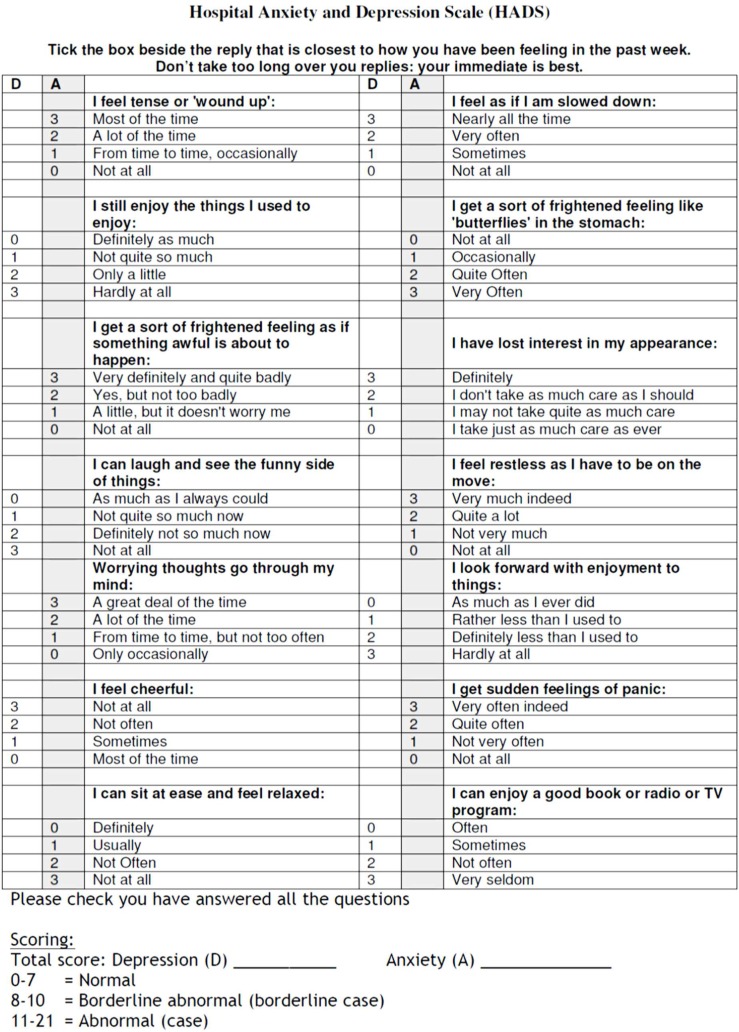
HAD scale questionnaire

Pain severity was again measured on the 100-mm VAS and was recorded. Instruction for using VAS and a questionnaire was given to the patients before medication. Patients were asked to record their pain score before administration of medicine and 10 min after local anesthesia. Furthermore postoperative pain was written down at 8, 12 and 24 h after endodontic treatment. Also the patient completed Hospital and Anxiety Depression (HAD) scale questionnaire [17] for evaluating their depression and anxiety effect on postoperative endodontic pain (Figure 1). Patients were also asked to record the possible side effects of the taken medicine and were assigned to record the consumption of additional analgesics. 

The data were collected and statistically analyzed using the chi-square, ANOVA, paired T-test, Tamhane and Pearson correlation coefficient using the SPSS software (Statistical Package for Social Science, SPSS, version 18.0, SPSS, Chicago, IL, USA). The level of significance was set at 0.05.

## Results

Table 1 shows the patients’ demographic data. There were no significant differences in distribution of age (*P*=0.36), gender (*P*=0.17), background depression (*P*=0.29) and anxiety (*P*=0.11). 

ANOVA analysis showed a significant difference in pain before endodontic treatment and 8 h after treatment (*P*=0.000) but there were no significant differences between the groups before treatment (*P*=0.67), and 12 h (*P*=0.80) and 24 h (*P*=0.27) after treatment.

**Figure 2:**
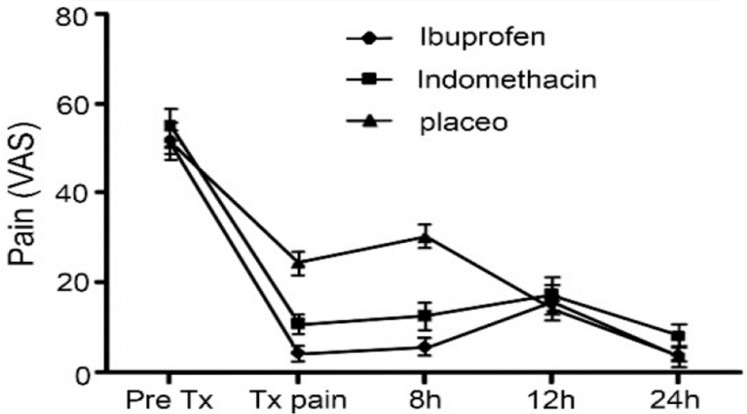
Mean time-related pain scores recorded on VAS

In fact, in ibuprofen group the severity of pain decreased after 8 and 12 h (*P*=0.23), whereas there were no significant differences in amount of pain in indomethacin group (*P*=0.14). Also in placebo group the severity of pain significantly increased (*P*=0.001). Also pairwise comparisons revealed that during the time of operation and 8 h after treatment ibuprofen (*P*<0.001) and indomethacin (*P*=0.001) groups had significantly lower pain scores than placebo. Mean VAS scores were plotted in relation to time after administration of the medicine (Figure 2).

## Discussion

Postoperative pain initiates a few hours after root canal therapy [17]. For relieving this pain, patients need analgesics and dentists should be able to prevent it. Pain control during and after endodontic treatment is one of the most important issues in endodontics [18].

Post-endodontic pain is often attributed to the inflammatory process as well as additional central mechanisms [19, 20]. Endodontic pain is noticeably different from pain induced by other dental procedures because of the presence of inflammation and pain before treatment [14]. Neuroplastic changes in the medullary dorsal horn can be caused by preexisting pulpal pain following acute inflammation related to anatomic structures [21]. 

A 5-fold increase in the discharge rate of dorsal horn neuron and up to a 3-fold increase in the size of the receptive field of *A-delta* fibers as a result of continuous peripheral nociceptive barrage from inflamed pulp have been shown in animal models [22, 23]. 

Postoperative pain is the result of periapical inflammatory reactions; so NSAIDs may be useful in controlling it [11]. Premedication with NSAIDs can reduce postoperative pain [10, 11, 13, 24, 25]. Some chemicals such as benzodiazepines, non-opioid analgesics and opioids have been utilized prophylactically to decrease post endodontic pain, among which, NSAIDS, especially ibuprofen have a noticeable role [26]. NSAIDs inhibit prostaglandin synthesis by decreasing the activity of the cyclooxygenase 1 and 2 (COX-1 and COX-2). Many NSAIDs like ibuprofen, aspirin [27], flurbiprofen [28], ketorolac [29, 30], and etodolac [31], have shown to produce a significant reduction in dental pain using clinical trials.

COX pathway can be blocked by prophylactic prescription of NSAIDs before treatment, so pain sensation will be reduced after RCT [13]. Because of this, investigators have found that preoperative prescription of NSAIDs decreased pain level a few hours after root canal therapy [10, 11, 13].

Ibuprofen safely blocks COX-1 and COX-2 enzymes and has analgesic and anti-inflammatory action [32]. For reduction of postoperative pain, 50 to 80 mg doses of ibuprofen should be used; however, Derry *et al.* [33] proposed that 200 and 400 mg ibuprofen had better efficacy.

Gopikrishna *et al. *[11] showed that single dose prophylactic prescription of rofecoxib (50 mg) or ibuprofen tablet (600 mg) significantly reduced postoperative endodontic pain. In another study by Arslan *et al.* [12] prophylactic administration of ibuprofen (200 mg) and tenoxicam (20 mg) significantly reduced the postoperative endodontic pain.

Clinical recommendation about NSAIDs was supported by systematic reviews [6, 34]. These drugs should be the medicine of choice in patients who can tolerate them [6].

Blood level of ibuprofen increase 1 to 2 h after ingestion and its absorption occurs immediately after oral administration [35]. Indomethacin as an NSAID has strong anti-inflammatory effects that are used for reduction of moderate to severe pain levels [15]. Generally indomethacin has not been used commonly in endodontic treatment. However, Parirokh *et al. *[16] have evaluated the effectiveness of indomethacin and ibuprofen on increasing the depth of inferior alveolar nerve block in molar teeth with irreversible pulpitis. 

So in this study, according to the strong analgesic effect of indomethacin, the probability of its effect on reduction of post-endodontic pain was assessed. But, all side effects of indomethacin should be considered before its prescription [15].

In this study the patients were monitored for 24 h. They didn^’^t report side effects. This may either show a reasonable case selection or that a single dose of either medication is unlikely to cause significant problems for the patients. 

**Table 1:** Distribution of patients in groups

**Group **	**Gender (%)**		**Mean (SD) of age (years)**	**Mean (SD) of depression**	**Mean (SD) of anxiety**
	**Male**	**Female**			
**Ibuprofen**	40.9	59.1	23.8 (2.9)	8.9 (1.8)	9.4 (1.7)
**Indomethacin**	31.8	68.2	23.8 (2.9)	8.5 (2.2)	8.2 (2.3)
**Placebo**	59.1	40.9	23.8 (2.9)	8 (1.8)	9.1 (1.7)
***P*** **-value**	0.179		0.364	0.293	0.114

Despite the previously mentioned efficacy of indomethacin, ibuprofen may be a better choice because it is not only a strong analgesic, but also has fewer side effects compared to indomethacin [6]. The analgesic effect of NSAIDs is dose-dependent and so are its side effects [6].

Our results showed that patients treated with ibuprofen and indomethacin significantly had lower pain ratings during treatment and at 8 h after treatment in comparison with placebo. However, the 12-h pain rating in these two groups were significantly higher than 8-h ratings. This could be attributed to the half-life of their metabolites, which is between 4 to 6 h. All groups gave similar pain ratings at 12 and 24 h after treatment.

Gospikrishna *et al. *[11] and Arslan *et al. *[12] declared that the analgesic effect of ibuprofen in comparison with placebo was significant at 4, 6 and 8 h after treatment while there were no significant differences after 12 h.

Due to the maximal analgesic effect of ibuprofen and indomethacin their effects did not last more than 8 h [11]. Moreover pain reduction 12 h after endodontic treatment in the placebo group may conclude that endodontic treatment combined with placebo medication may reduce pain 12 h after the initiation of treatment [12, 14]. These results emphasize on earlier investigations evaluating the reduction in pain after 12 h in patients undergone single visit endodontic treatment.

The measurement of pain is difficult because pain perception is subjective and variable which is regulated by multiple physical and psychological factors [36]. Many studies have suggested that pain conditions to be associated with self-reports of psychological distress and psychiatric disorders [37]. Also depression and anxiety symptoms are reported to be associated with reports of increased pain [38-42].

## Conclusion

Prophylactic administration of 400 mg ibuprofen in a single-dose provides effective reduction of post-operative pain lasting for 8 h.

## References

[1] Goodale DB (1982). Inhibition of substance P release is the key to successful management of oral pain. Anesth Prog.

[2] Ince B, Ercan E, Dalli M, Dulgergil CT, Zorba YO, Colak H (2009). Incidence of postoperative pain after single- and multi-visit endodontic treatment in teeth with vital and non-vital pulp. Eur J Dent.

[3] Pochapski MT, Santos FA, de Andrade ED, Sydney GB (2009). Effect of pretreatment dexamethasone on postendodontic pain. Oral Surg Oral Med Oral Pathol Oral Radiol Endod.

[4] Malmberg A, Yaksh T (1992). Hyperglycemia mediated by spinal glutamate or substance P receptor blocked by spinal cyclooxygenase inhibition. science.

[5] Torabinejad M, Bakland LK (1980). Prostaglandins: their possible role in the pathogenesis of pulpal and periapical diseases, part 1. J Endod.

[6] Hargreaves k, Abbott P (2005). Drugs for pain management in dentistry. Australian Dental Journal Medications Supplement.

[7] Vane J, Botting R (2005). The mechanism of action of aspirin. Thromb Res.

[8] Altinoz MA, Korkmaz R (2004). NF-kappaB, macrophage migration inhibitory factor and cyclooxygenase-inhibitions as likely mechanisms behind the acetaminophen- and NSAID-prevention of the ovarian cancer. Neoplasma.

[9] Fernandes E, Costa D, Toste SA, Lima JL, Reis S (2004). In vitro scavenging activity for reactive oxygen and nitrogen species by nonsteroidal anti-inflammatory indole, pyrrole, and oxazole derivative drugs. Free Radic Biol Med.

[10] Flath RK, Hicks ML, Dionne RA, Pelleu GB Jr (1987). Pain suppression after pulpectomy with preoperative flurbiprofen. J Endod.

[11] Gopikrishna V, Parameswaran A (2003). Effectiveness of prophylactic use of rofecoxib in comparison with ibuprofen on postendodontic pain. J Endod.

[12] Arslan H, Topcuoglu HS, Aladag H (2011). Effectiveness of tenoxicam and ibuprofen for pain prevention following endodontic therapy in comparison to placebo: a randomized double-blind clinical trial. J Oral Sci.

[13] Menke ER, Jackson CR, Bagby MD, Tracy TS (2000). The effectiveness of prophylactic etodolac on postendodontic pain. J Endod.

[14] Attar S, Bowles WR, Baisden MK, Hodges JS, McClanahan SB (2008). Evaluation of pretreatment analgesia and endodontic treatment for postoperative endodontic pain. J Endod.

[15] Legrand E (2004). Aceclofenac in the management of inflammatory pain. Expert Opin Pharmacother.

[16] Parirokh M, Ashouri R, Rekabi AR, Nakhaee N, Pardakhti A, Askarifard S, Abbott PV (2010). The effect of premedication with ibuprofen and indomethacin on the success of inferior alveolar nerve block for teeth with irreversible pulpitis. J Endod.

[17] Zigmond AS, Snaith RP (1983). The hospital anxiety and depression scale. Acta Psychiatr Scand.

[18] Tinastepe N, Oral K (2013). Neuropathic pain after dental treatment. Agri.

[19] Atbaei A, Mortazavi N (2012). Prophylactic intraligamentary injection of piroxicam (feldene) for the management of post-endodontic pain in molar teeth with irreversible pulpitis. Aust Endod J.

[20] Parirokh M, Rekabi AR, Ashouri R, Nakhaee N, Abbott PV, Gorjestani H (2013). Effect of occlusal reduction on postoperative pain in teeth with irreversible pulpitis and mild tenderness to percussion. J Endod.

[21] Torneck CD, Kwan CL, Hu JW (1996). Inflammatory lesions of the tooth pulp induce changes in brainstem neurons of the rat trigeminal subnucleus oralis. J Dent Res.

[22] Chiang CY, Park SJ, Kwan CL, Hu JW, Sessle BJ (1998). NMDA receptor mechanisms contribute to neuroplasticity induced in caudalis nociceptive neurons by tooth pulp stimulation. J Neurophysiol.

[23] Närhi M, Ngassapa D, Shimono MMT, Suda H, Takahashi K (1996). Function of intradental nociceptors in normal and inflamed teeth In Dentin/pulp complex. Tokyo:Quintessence.

[24] Dionne RA, Campbell RA, Cooper SA, Hall DL, Buckingham B (1983). Suppression of postoperative pain by preoperative administration of ibuprofen in comparison to placebo, acetaminophen, and acetaminophen plus codeine. J Clin Pharmacol.

[25] Dionne RA, Cooper SA (1978). Evaluation of preoperative ibuprofen for postoperative pain after removal of third molars. Oral Surg Oral Med Oral Pathol.

[26] Kanaa MD, Whitworth JM, Meechan JG (2012). A prospective randomized trial of different supplementary local anesthetic techniques after failure of inferior alveolar nerve block in patients with irreversible pulpitis in mandibular teeth. J Endod.

[27] Holstein A, Hargreaves KM, Niederman R (2002). Evaluation of NSAIDs for treating post-endodontic pain. Endod Topics.

[28] Doroschak AM, Bowles WR, Hargreaves KM (1999). Evaluation of the combination of flurbiprofen and tramadol for management of endodontic pain. J Endod.

[29] Mehrvarzfar P, Abbott PV, Saghiri MA, Delvarani A, Asgar K, Lotfi M, Karamifar K, Kharazifard MJ, Khabazi H (2012). Effects of three oral analgesics on postoperative pain following root canal preparation: a controlled clinical trial. Int Endod J.

[30] Penniston SG, Hargreaves KM (1996). Evaluation of periapical injection of Ketorolac for management of endodontic pain. J Endod.

[31] Comfort MB, Tse AS, Tsang AC, McGrath C (2002). A study of the comparative efficacy of three common analgesics in the control of pain after third molar surgery under local anaesthesia. Aust Dent J.

[32] Richy F, Bruyere O, Ethgen O, Rabenda V, Bouvenot G, Audran M, Herrero-Beaumont G, Moore A, Eliakim R, Haim M, Reginster JY (2004). Time dependent risk of gastrointestinal complications induced by non-steroidal anti-inflammatory drug use: a consensus statement using a meta-analytic approach. Ann Rheum Dis.

[33] Derry C, Derry S, Moore RA, McQuay HJ Single dose oral ibuprofen for acute postoperative pain in adults. Cochrane Database Syst Rev.

[34] Sutherland S, Matthews DC (2003). Emergency management of acute apical periodontitis in the permanent dentition: a systematic review of the literature. J Can Dent Assoc.

[35] Read JK, McClanahan SB, Khan AA, Lunos S, Bowles WR (2014). Effect of Ibuprofen on masking endodontic diagnosis. J Endod.

[36] DiRenzo A, Gresla T, Johnson BR, Rogers M, Tucker D, BeGole EA (2002). Postoperative pain after 1- and 2-visit root canal therapy. Oral Surg Oral Med Oral Pathol Oral Radiol Endod.

[37] McWilliams LA, Goodwin RD, Cox BJ (2004). Depression and anxiety associated with three pain conditions: results from a nationally representative sample. Pain.

[38] Carroll LJ, Cassidy JD, Cote P (2004). Depression as a risk factor for onset of an episode of troublesome neck and low back pain. Pain.

[39] Casten RJ, Parmelee PA, Kleban MH, Lawton MP, Katz IR (1995). The relationships among anxiety, depression, and pain in a geriatric institutionalized sample. Pain.

[40] Doan BD, Wadden NP (1989). Relationships between depressive symptoms and descriptions of chronic pain. Pain.

[41] Leino P, Magni G (1993). Depressive and distress symptoms as predictors of low back pain, neck-shoulder pain, and other musculoskeletal morbidity: a 10-year follow-up of metal industry employees. Pain.

[42] Vaeroy H, Tanum L, Bruaset H, Morkrid L, Forre O (2005). Symptoms of depression and anxiety in functionally disabled rheumatic pain patients. Nord J Psychiatry.

